# Supplementary material for: ABHD4-Regulating RNA Panel: Novel Biomarkers in Acute Coronary Syndrome Diagnosis
Source: Cells. 2021 Jun 16;10(6):1512. doi: 10.3390/cells10061512 (PMC8235602; doi:10.3390/cells10061512)
Supplement: Supplementary file 1 [file cells-10-01512-s001.zip › cells-1230233-supplementary.pdf]

cells

MDPI

Article

ABHD4-Regulating RNA Panel: Novel Biomarkers in Acute Coronary Syndrome Diagnosis

Sara H.A. Agwa [1,*], Sherif Samir Elzahwy [2], Mahmoud Shawky El Meteini [3], Hesham Elghazaly [4], Maha Saad [5], Aya M. Abd Elsamee [6], Rania Shamekh [7] and Marwa Matboli [8,*]

[1] Clinical pathology and Molecular Genomics Unit, Medical Ain Shams Research Institute (MASRI), Faculty of Medicine, Ain Shams University, Cairo 11382, Egypt
[2] Cardiovascular Medicine Department, Faculty of Medicine, Ain Shams University, Cairo 1382, Egypt; Dr.sherifelzahwy@med.asu.edu.eg
[3] Department of General Surgery, the School of Medicine, University of Ain Shams, Cairo 11382, Egypt; mahmoud_elmeteini@med.asu.edu.eg
[4] Oncology Department, Medical Ain Shams Research institute (MASRI), Faculty of Medicine, Ain Shams University, Cairo 11382, Egypt; heshamelghazaly@med.asu.edu.eg
[5] Biochemistry Department, Faculty of Medicine, Modern University for Technology and Information, Cairo 11382, Egypt; maha.saad@medicine.mti.edu.eg
[6] Biochemistry and Molecular Genomics Unit, Medical Ain Shams Research Institute (MASRI), Faculty of Medicine, Ain Shams University, Cairo 11382, Egypt; aya_ana2025@yahoo.com
[7] Department of Pathology, University of South Florida, Tampa, FL 33620, USA; Rania.shamekh@gmail.com
[8] Medicinal Biochemistry and Molecular Biology Department, Faculty of Medicine, Ain Shams University, Cairo 11382, Egypt
* Correspondence: sara.h.agwa@med.asu.edu.eg (S.H.A.A.); drmarwa_matboly@med.asu.edu.eg (M.M.)

Methodology Details

We have used the following products: U6 sn RNA, TaqMan™ microRNA Control Assay 5X (RT: 001973), hsa-miR-221 TaqMan™ Advanced miRNA Assay 5X (RT: 00052), hsa-miR-197 TaqMan™ Advanced miRNA Assay 5X (RT: 000497), ABHD4 TaqMan™ Gene Expression Assay (FAM) (HS01040459-m1) (Thermo Fisher Scientific, Inc,Waltham, MA, USA), Human FENDRR RT2 lncRNA qPCR Assay (LPH26005A-200), and Hs-ACTB-1-RT2 QuantiTect Primer Assays (Cat. No. QT00095431) (Qiagen, Germany).

Table S1. Study Population Demographic and Clinical Characteristics (n = 110).

	**Acute myocardial infarction**	**Non-cardiac chest pain**	**Control**
Overall	68	21	21
Type of ACS			
Unstable angina (n = 21)	21 (30.9%)	0%	0%
STEMI (n = 31)	31 (45.6%)	0%	0%
Non STEMI (n = 16	16 (23.5)	0%	0%
χ^2 (p)			
Age<br>Mean ± S.D	55.7 ± 11.7	56.7 ± 10.7	54.6 ± 10.2
F(p)		.225 (.779)	
Sex			
Male (n = 87)	54 (79.4%)	16 (72.2%)	17 (81%)
Female (n = 23)	14 (20.6%)	5 (23.8%)	4 (19%)
χ^2 (p)		.155 (0.925)	
Smoking			15 (71.4%)

Positive (n = 75)	47 (69.1%)	13 (61.9%)	6 (28.6%)
Negative (n = 35)	21 (30.9%)	8 (38.1.5%)	
χ^2 (p)		.511 (0.75)	
Hypertension			
Positive (n = 34)	23 (33.8%)	5 (23.8%)	6 (28.6%)
Negative (n = 76)	45 (66.2%)	16(67.2%)	15 (71.4%)
χ^2(P)		.820 (0.664)	
Diabetes Mellitus			
Positive (n = 38)	24 (35.5%)	5 (23.8%)	9 (42.9%)
Negative (n = 72)	4 4(64.7%)	16 (67.2%)	12 (56.1%)
χ^2 (p)		1.729 (0.421)	
Onset of chest pain Mean ± S.D	6.8 ± 1.077	6.22 ± 1.023	0.00
F (p)		0.604 (.523)	
Body mass index Mean ± S.D	27.2 ± 3.9	26.6 ± 2.6	25.4 ± 1.7
F (p)		5.03 (0.008)	
TG (mg/dL) Mean ± S.D	132.09 ± 44.66	66 ± 29.04	71.1 ± 28.8
F (p)		33.5 (0.000)	
HDL (mg/dL) Mean ± S.D	31.7 ± 6.4	33.2 ± 7.5	33.4 ± 9.3
F (p)		0.671(0.53)	
LDL (mg/dL) Mean ± S.D	128.19±28.3	119.8 ± 25.5	124.7 ± 28.2
F (p)		1.35 (0.262)	
Cholesterol (mg/dL) Mean ± S.D	211.9±33.2	201.19 ± 32.3	201.4 ± 28.3
F (p)		1.408 (0.249)	
Serum Creatinine (mg/dL) Mean ± S.D	1.827±1.9	0.95 ± 0.38	1.1 ± 0.3
F (p)		3.04 (0.037)	

S.D.: Standard Deviation, χ2: Chi-Square test, p: level of significance, F: One Way Anova test.

	SuperPathway			Contained pathways	
1	**Glycerophospholipid biosynthesis**	Glycerophospholipid biosynthesis	0.63	Phospholipid metabolism	0.63
2	**Metabolism**	Metabolism	0.40	Metabolism of lipids and lipoproteins	0.37
3	**Acyl chain remodelling of PE**	Acyl chain remodelling of PE	0.70		

GenesLikeMe Genes that share pathways with ABHD4: view

Pathways by source for ABHD4 Gene

5 Reactome pathways for ABHD4 Gene

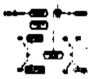

- Acyl chain remodelling of PE
- Glycerophospholipid biosynthesis
- Metabolism
- Metabolism of lipids and lipoproteins
- Phospholipid metabolism

Gene Ontology (GO) - Biological Process for ABHD4 Gene

Filter: *(6 results)* See all 6 »

GO ID	Qualified GO term	Evidence
GO:0006629	lipid metabolic process	IEA
GO:0006654	phosphatidic acid biosynthetic process	IBA
GO:0016042	lipid catabolic process	IEA
GO:0036152	phosphatidylethanolamine acyl-chain remodeling	TAS
GO:0055088	lipid homeostasis	IBA

Function for ABHD4 Gene

Products: Animal Model / CRISPR / miRNA / Inhibitory RNA / Clone / Cell Line

Molecular function for ABHD4 Gene

UniProtKB/Swiss-Prot Function:	Lysophospholipase selective for N-acyl phosphatidylethanolamine (NAPE). Contributes to the biosynthesis of N-acyl ethanolamines, including the endocannabinoid anandamide by hydrolyzing the sn-1 and sn-2 acyl chains from N-acyl phosphatidylethanolamine (NAPE) generating glycerophospho-N-acyl ethanolamine (GP-NAE), an intermediate for N-acyl ethanolamine biosynthesis. Hydrolyzes substrates bearing saturated, monounsaturated, polyunsaturated N-acyl chains. Shows no significant activity towards other lysophospholipids, including lysophosphatidylcholine, lysophosphatidylethanolamine and lysophosphatidylserine. ABHD4_HUMAN,Q8TB40
UniProtKB/Swiss-Prot CatalyticActivity:	Reaction=H2O + N-hexadecanoyl-1,2-di-(9Z-octadecenoyl)-sn-glycero-3-phosphoethanolamine = (9Z)-octadecenoate + H(+) + N-hexadecanoyl-1-(9Z-octadecenoyl)-sn-glycero-3-phosphoethanolamine; Xref=Rhea:RHEA:45424, ChEBI:CHEBI:15377, ChEBI:CHEBI:15378, ChEBI:CHEBI:30823, ChEBI:CHEBI:78097, ChEBI:CHEBI:85217; Evidence=. ; PhysiologicalDirection=left-to-right; Xref=Rhea:RHEA:45425; Evidence=. ;. ABHD4_HUMAN,Q8TB40
UniProtKB/Swiss-Prot CatalyticActivity:	Reaction=H2O + N-acyl-1,2-diacyl-sn-glycero-3-phosphoethanolamine = a fatty acid + H(+) + N,1-diacyl-sn-glycero-3-phosphoethanolamine; Xref=Rhea:RHEA:45460, ChEBI:CHEBI:15377, ChEBI:CHEBI:15378, ChEBI:CHEBI:28868, ChEBI:CHEBI:62537, ChEBI:CHEBI:85216; Evidence=. ; PhysiologicalDirection=left-to-right; Xref=Rhea:RHEA:45461; Evidence=. ;. ABHD4_HUMAN,Q8TB40
UniProtKB/Swiss-Prot CatalyticActivity:	Reaction=H2O + N-hexadecanoyl-1-(9Z-octadecenoyl)-sn-glycero-3-phosphoethanolamine = (9Z)-octadecenoate + H(+) + N-hexadecanoyl-sn-glycero-3-phosphoethanolamine; Xref=Rhea:RHEA:45384, ChEBI:CHEBI:15377, ChEBI:CHEBI:15378, ChEBI:CHEBI:30823, ChEBI:CHEBI:85217, ChEBI:CHEBI:85226; Evidence=. ; PhysiologicalDirection=left-to-right; Xref=Rhea:RHEA:45385; Evidence=. ;. ABHD4_HUMAN,Q8TB40
UniProtKB/Swiss-Prot CatalyticActivity:	Reaction=H2O + N-octadecanoyl-1-(9Z-octadecenoyl)-sn-glycero-3-phosphoethanolamine = (9Z)-octadecenoate + H(+) + N-octadecanoyl-sn-glycero-3-phospho-ethanolamine; Xref=Rhea:RHEA:45388, ChEBI:CHEBI:15377, ChEBI:CHEBI:15378, ChEBI:CHEBI:30823, ChEBI:CHEBI:85219, ChEBI:CHEBI:85227; Evidence=. ; PhysiologicalDirection=left-to-right; Xref=Rhea:RHEA:45389; Evidence=. ;. ABHD4_HUMAN,Q8TB40
UniProtKB/Swiss-Prot CatalyticActivity:	Reaction=H2O + N-eicosanoyl-1-(9Z-octadecenoyl)-sn-glycero-3-phosphoethanolamine = (9Z)-octadecenoate + H(+) + N-eicosanoyl-sn-glycero-3-phosphoethanolamine; Xref=Rhea:RHEA:45392, ChEBI:CHEBI:15377, ChEBI:CHEBI:15378, ChEBI:CHEBI:30823, ChEBI:CHEBI:85221, ChEBI:CHEBI:85228; Evidence=. ; PhysiologicalDirection=left-to-right; Xref=Rhea:RHEA:45393; Evidence=. ;. ABHD4_HUMAN,Q8TB40
UniProtKB/Swiss-Prot CatalyticActivity:	Reaction=H2O + N,1-di-(9Z-octadecenoyl)-sn-glycero-3-phosphoethanolamine = (9Z)-octadecenoate + H(+) + N-(9Z-octadecenoyl)-sn-glycero-3-phosphoethanolamine; Xref=Rhea:RHEA:45396, ChEBI:CHEBI:15377, ChEBI:CHEBI:15378, ChEBI:CHEBI:30823, ChEBI:CHEBI:85222, ChEBI:CHEBI:85229; Evidence=. ; PhysiologicalDirection=left-to-right; Xref=Rhea:RHEA:45397; Evidence=. ;. ABHD4_HUMAN,Q8TB40

Activate Windows
Go to Settings to activate W

Figure S1. Gene ontology of ABHD4 mRNA by KEGG map that was retrieved from GeneCards®: The Human Gene Database. It shows that ABHD4 mRNA is linked specifically to phospholipid metabolism. Available at (https://www.genecards.org/cgi-bin/carddisp.pl?gene=ABHD4, accessed on 21 May 2021)).

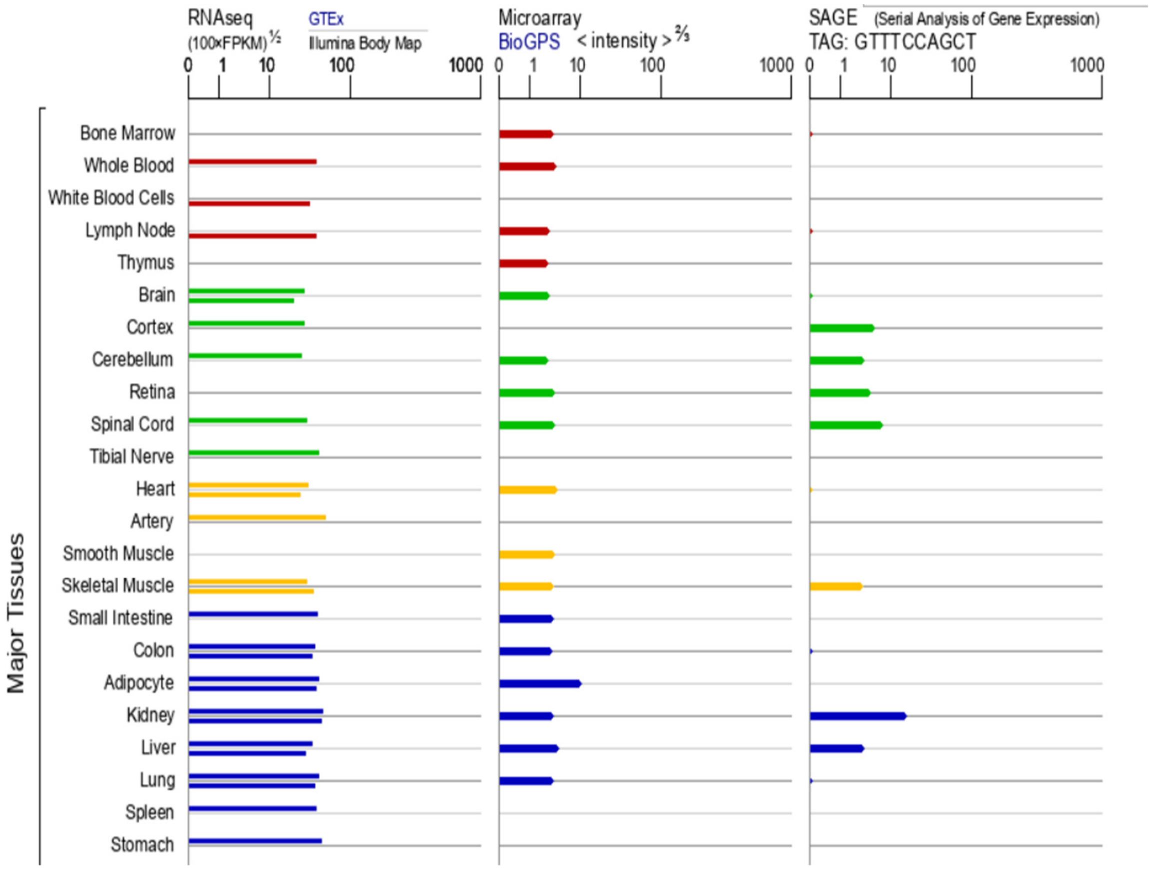

Figure S2: Gene expression of ABHD4 mRNA in heart that was retrieved from GeneCards®: The Human Gene Database. It shows basal RNA expression in normal heart. Available at (https://www.genecards.org/cgi-bin/carddisp.pl?gene=ABHD4, (accessed on 27 May 2021)). X-axis represents level of RNA expression while Y-axis represents the tissue in which the gene is expressed.

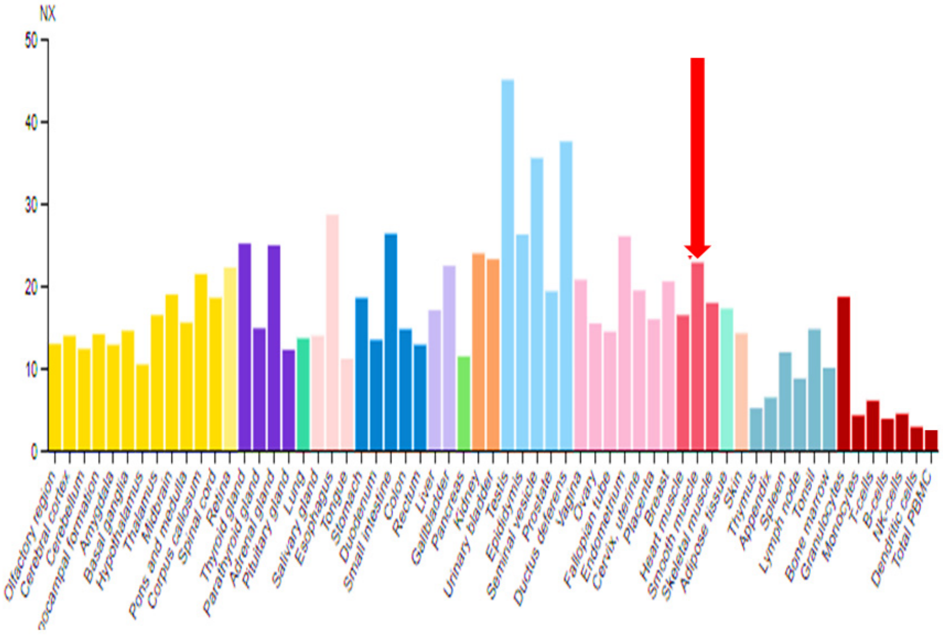

Figure S3. Gene expression of ABHD4 mRNA in heart that was retrieved from The Human Protein Atlas database. It shows the basal expression of ABHD4 mRNA in heart. The red arrow refers to the level of ABHD4 mRNA expression in normal heart. Y-axis represents level of RNA expression while X-axis represents the tissue in which the gene is expressed.

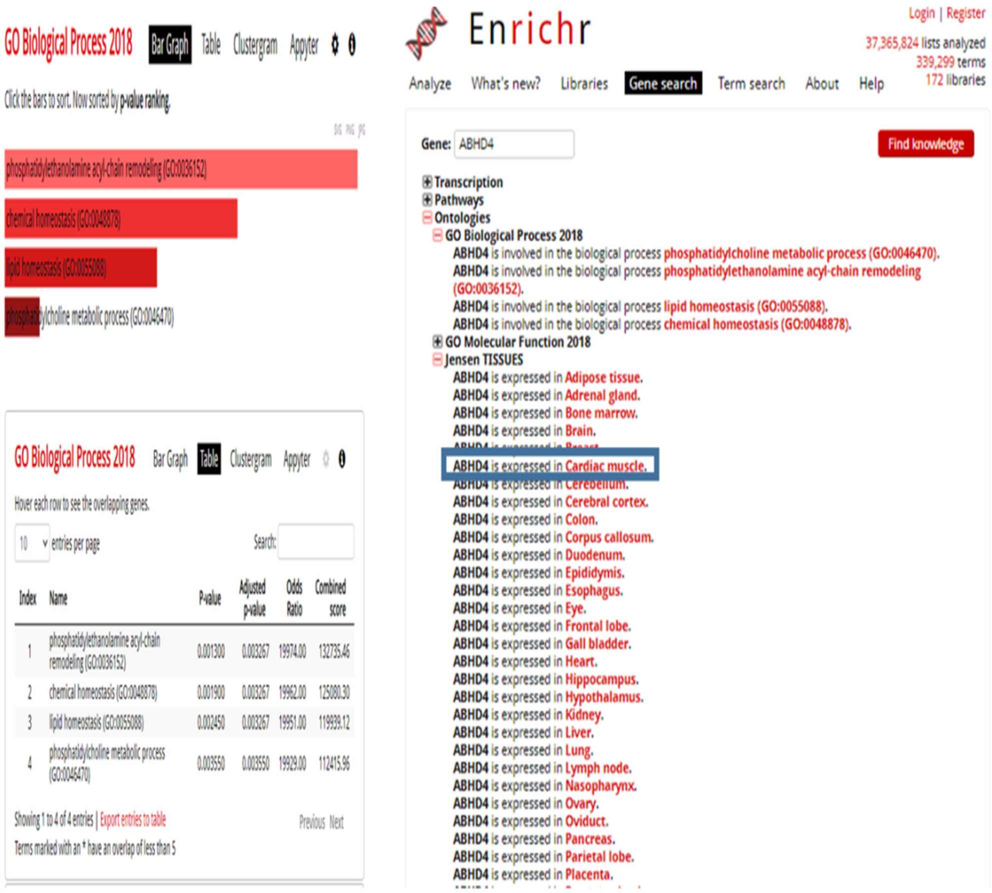

Figure S4. Gene ontology and expression of ABHD4 mRNA in heart that was retrieved from Enricher tool (https://maayanlab.cloud/Enrichr/ (accessed on 1 june 2021)). It shows the expression of ABHD4 mRNA in heart and its link to phospholipid metabolism.

	SuperPathway	Contained pathways			
1	**ADP signalling through P2Y purinoceptor 12**	ADP signalling through P2Y purinoceptor 12<br>G-protein activation	0.73<br>0.73	Cooperation of PDCL (PhLP1) and TRiC/CCT in G-protein beta folding<br>G alpha (z) signalling events	0.57<br>0.45
2	**DAG and IP3 signaling**	PLC beta mediated events<br>G-protein mediated events	0.65<br>0.64	Opioid Signalling<br>G Protein Signaling Pathways	0.56<br>0.44
3	**GABA receptor activation**	Activation of GABAB receptors<br>GABA B receptor activation	0.71<br>0.71	GABA receptor activation	0.71
4	**Signaling by GPCR**	GPCR downstream signaling<br>Signaling by GPCR	0.76<br>0.76	Signal Transduction	0.52
5	**Transmission across Chemical Synapses**	Neurotransmitter Receptor Binding And Downstream Transmission In The Postsynaptic Cell<br>Transmission across Chemical Synapses	0.67<br>0.67	Neuronal System	0.62

Gene Ontology (GO) - Biological Process for MIR197 Gene

Filter: (3 results)

GO ID	Qualified GO term	Evidence
GO:0032701	negative regulation of interleukin-18 production	IDA
GO:0035195	gene silencing by miRNA	IDA,IEA
GO:1900015	regulation of cytokine production involved in inflammatory response	IDA

Figure S5. Pathway enrichment analysis of miR-197 by KEGG map that was retrieved from GeneCards®: The Human Gene Database (https://www.genecards.org/cgi-bin/carddisp.pl?gene=MIR197 (accessed on 25 May 2021)). It clarifies the role of miRNA-197 in inflammatory response and diacyl glyceol (DAG) and lipid metabolism that is closely linked to myocardial ischemia.

Items 1 - 25 of 45 < Prev Page 1 of 2 Next >

Process	Evidence Code	Pubs
gene silencing by miRNA	IDA	PubMed
gene silencing by miRNA	IEA	
gene silencing by miRNA	IGI	PubMed
gene silencing by miRNA	IMP	PubMed
miRNA mediated inhibition of translation	IDA	PubMed
miRNA mediated inhibition of translation	IMP	PubMed
negative regulation by host of viral genome replication	ISS	
negative regulation of ERK1 and ERK2 cascade	IDA	PubMed
negative regulation of TRAIL-activated apoptotic signaling pathway	IDA	PubMed
negative regulation of TRAIL-activated apoptotic signaling pathway	IMP	PubMed
negative regulation of apoptotic process	IDA	PubMed
negative regulation of apoptotic process	IMP	PubMed
negative regulation of cell adhesion molecule production	IGI	PubMed
negative regulation of cell migration involved in sprouting angiogenesis	IGI	PubMed
negative regulation of cell population proliferation	IDA	PubMed
negative regulation of cytokine production involved in inflammatory response	ISS	
negative regulation of double-strand break repair	IDA	PubMed
negative regulation of hematopoietic stem cell proliferation	IDA	PubMed
negative regulation of heterotypic cell-cell adhesion	IDA	PubMed
negative regulation of inflammatory response	IGI	PubMed
negative regulation of interleukin-21 production	IDA	PubMed
negative regulation of leukocyte adhesion to vascular endothelial cell	IGI	PubMed
negative regulation of necroptotic process	IDA	PubMed
negative regulation of sprouting angiogenesis	IGI	PubMed
negative regulation of vascular associated smooth muscle cell differentiation	IMP	PubMed

Figure S6. Pathway enrichment analysis of miR-221-3p by KEGG map that was retrieved from https://www.ncbi.nlm.nih.gov/gene/407006 (accessed on 28 May 2021). It clarifies the role of miRNA-221 in inflammatory response and apoptosis that is closely linked to myocardial ischemia.

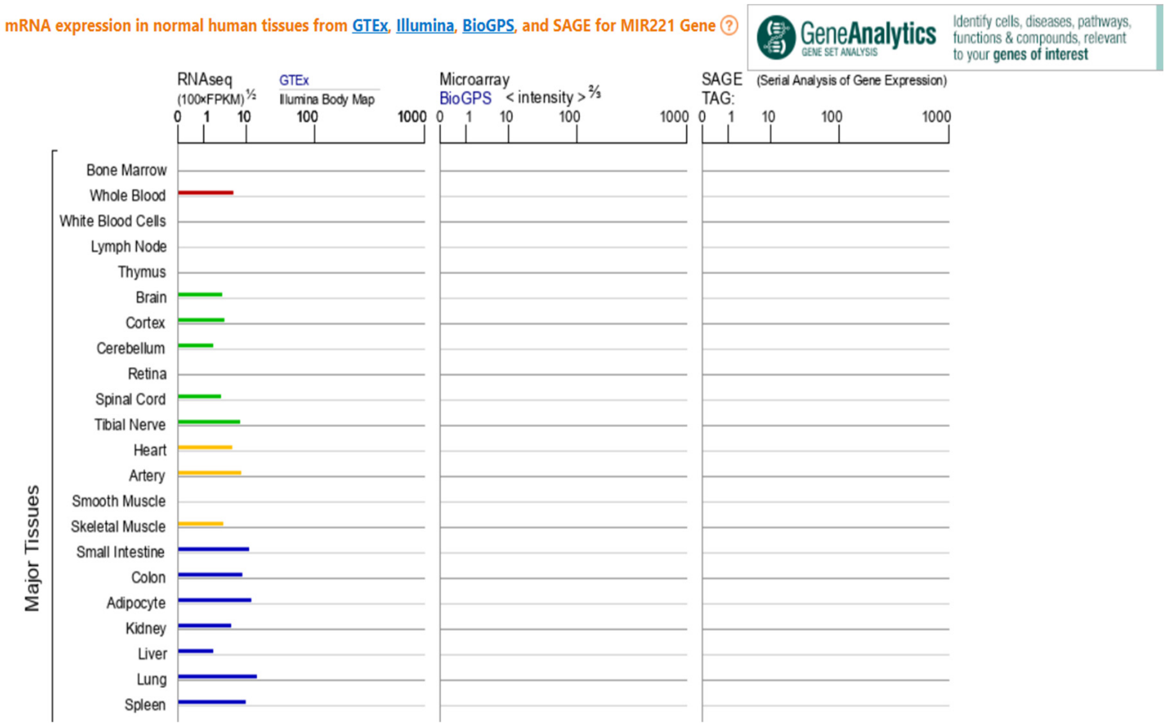

Figure S7: Gene expression of miRNA-221-3p in heart that was retrieved from GeneCards®: The Human Gene Database. Available at (https://www.genecards.org/cgi-bin/carddisp.pl?gene=MIR221 (accessed on 1 June 2021). It shows the basal expression of miRNA-221 in normal heart. X-axis represents level of RNA expression while Y-axis represents the tissue in which the gene is expressed.

Mirna	Refseqid	Genesymbol	Score	Position	Binding Site	Au	Me	N Pairings	Targetscan	Mirdb	Mirtarbase
hsa-miR-197-5p	NM_022060	ABHD4	0.92	3UTR	2221,2257	0.41	-5.036	20	–	–	–
hsa-miR-197-5p	NM_022060	ABHD4	0.85	3UTR	1144,1168	0.52	-6.937	19	–	–	–
hsa-miR-197-5p	NM_022060	ABHD4	1.00	3UTR	1994,2016	0.43	-9.552	18	–	–	–
hsa-miR-197-3p	NM_022060	ABHD4	1.00	3UTR	2047,2070	0.41	-9.547	17	–	–	–
hsa-miR-198	NM_022060	ABHD4	1.00	3UTR	1425,1445	0.46	-5.083	18	–	–	–
hsa-miR-198	NM_022060	ABHD4	1.00	3UTR	1991,2015	0.43	-5.763	18	–	–	–
hsa-miR-199a-5p	NM_022060	ABHD4	1.00	3UTR	2341,2362	0.34	-6.983	19	–	–	–
hsa-miR-208a-5p	NM_022060	ABHD4	0.92	5UTR	1,43	0.34	-3.938	16	–	–	–
hsa-miR-208a-5p	NM_022060	ABHD4	1.00	CDS	632,649	0.4	-9.22	16	–	–	–
hsa-miR-129-5p	NM_022060	ABHD4	0.92	3UTR	1743,1762	0.46	-15.567	15	–	–	–

Figure S8: ABHD4 mRNA is a direct target of miRNA-197-5p that was retrieved from miRWalk database (accessed on 1 june 2020). It shows direct interaction between miRNA-197-5p and ABHD4 mRNA.

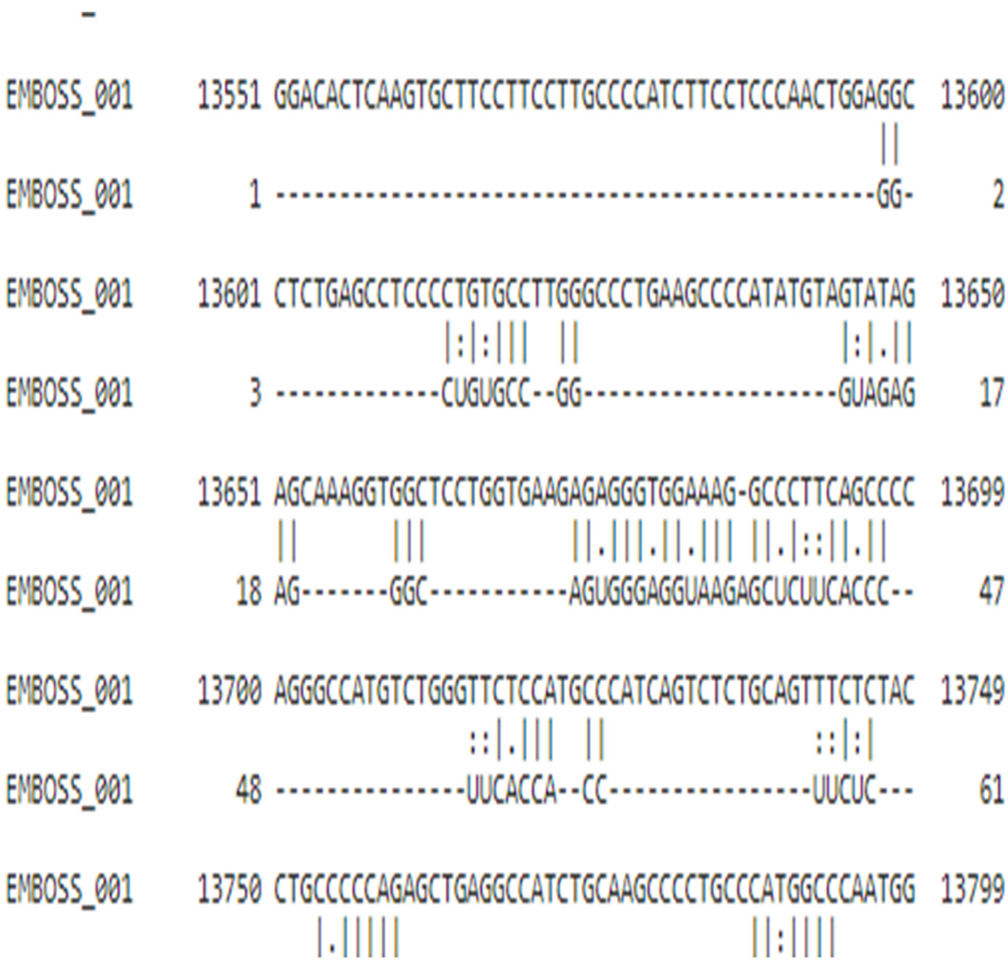

Figure S9. Alignment between ABHD4 mRNA and miRNA-197-5p that was performed using European bioinformatics institute database (accessed on 1 January 2021). It shows direct interaction between miRNA-197-5p and ABHD4 mRNA.

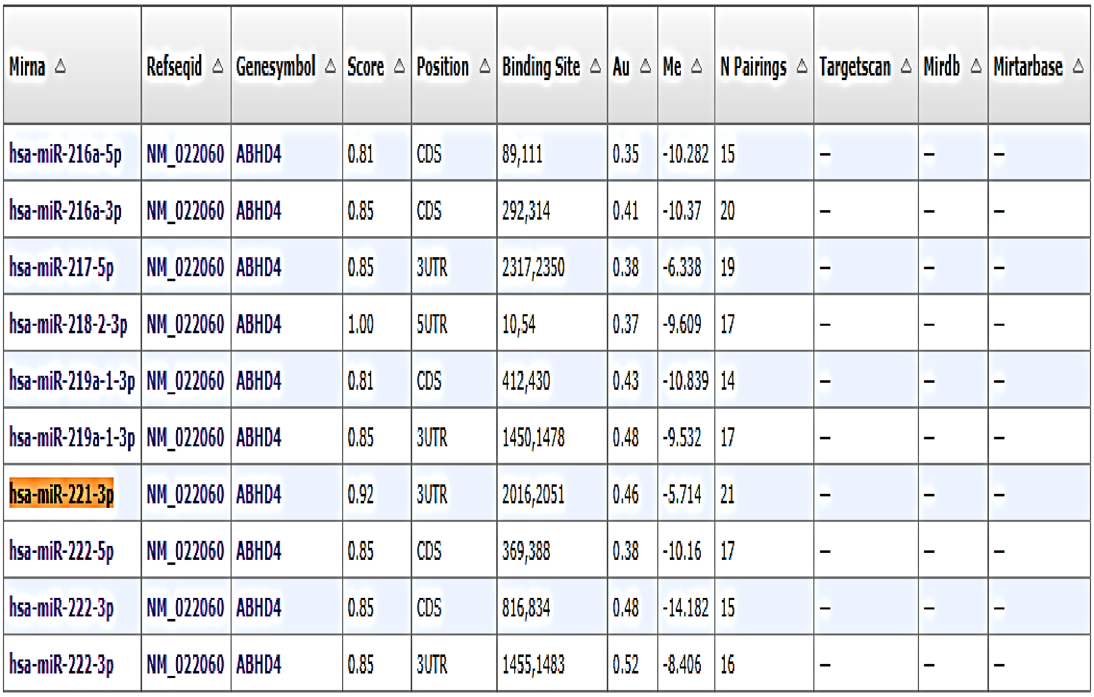

Mirna	Refseqid	Genesymbol	Score	Position	Binding Site	Au	Me	N Pairings	Targetscan	Mirdb	Mirtarbase
hsa-miR-216a-5p	NM_022060	ABHD4	0.81	CDS	89,111	0.35	-10.282	15	–	–	–
hsa-miR-216a-3p	NM_022060	ABHD4	0.85	CDS	292,314	0.41	-10.37	20	–	–	–
hsa-miR-217-5p	NM_022060	ABHD4	0.85	3UTR	2317,2350	0.38	-6.338	19	–	–	–
hsa-miR-218-2-3p	NM_022060	ABHD4	1.00	5UTR	10,54	0.37	-9.609	17	–	–	–
hsa-miR-219a-1-3p	NM_022060	ABHD4	0.81	CDS	412,430	0.43	-10.839	14	–	–	–
hsa-miR-219a-1-3p	NM_022060	ABHD4	0.85	3UTR	1450,1478	0.48	-9.532	17	–	–	–
hsa-miR-221-3p	NM_022060	ABHD4	0.92	3UTR	2016,2051	0.46	-5.714	21	–	–	–
hsa-miR-222-5p	NM_022060	ABHD4	0.85	CDS	369,388	0.38	-10.16	17	–	–	–
hsa-miR-222-3p	NM_022060	ABHD4	0.85	CDS	816,834	0.48	-14.182	15	–	–	–
hsa-miR-222-3p	NM_022060	ABHD4	0.85	3UTR	1455,1483	0.52	-8.406	16	–	–	–

Figure S10: ABHD4 mRNA is a direct target of miRNA-221-3p that was retrieved from miRWalk database. (accessed on 1 June 2021). It shows direct interaction between miRNA-221-3p and ABHD4 mRNA.

```
EMBOSS_001         1 --------------------------------------------------      0

EMBOSS_001      8301 GGGCCGCTCCCCAGGCCAGCTTGGGGCAGACAGTTGTTAGGAGATAAAAC   8350
                                      |||:        ||| ::|:             
EMBOSS_001         1 -----------------AGCU--------ACA-UUGU-------------     11

EMBOSS_001      8351 TCTTAGCTGCTGGAGTTCTGGGCTTTATTTCTTCTTCAACAAAGCTCTGA   8400
                           |:||:||..::|                                
EMBOSS_001        12 ------CUGCUGGGUUUC--------------------------------     23

EMBOSS_001      8401 CTCAGTTAGGTAAACACAGGCACTAGCAAGCCTGATCATTTCCAGAGAGT   8450

EMBOSS_001        24 --------------------------------------------------     23

EMBOSS_001      8451 TCCCCAGGGACAGACACAAAGGAATACAGAAGATAATAGATTAATTAAAC   8500

EMBOSS_001        24 --------------------------------------------------     23

EMBOSS_001      8501 ATGCTGCCCATCTCTAGAGGAAGGCCCTCCCTGTTCCCTACAGGTATGCA   8550

EMBOSS_001        24 --------------------------------------------------     23

EMBOSS_001      8551 ATTGGCCTGATGAACAAGAGTCTGGAAAATATTAATATTTTAAAAGATTT   8600

EMBOSS_001        24 --------------------------------------------------     23

EMBOSS_001      8601 TTTTAACTGAAAAATTGATACAAACATGGTATTTTTTTTCTTTTTTTAGG   8650

EMBOSS_001        24 --------------------------------------------------     23

EMBOSS_001      8651 TCAAAAATAATTAGCAAAAACATGATATTTTTCAAAAGGAAAGGAAGTCT   8700
```

Figure S11. Alignment between ABHD4 mRNA and miRNA-221-3p that was performed using European bioinformatics institute database (accessed on 1 June 2021). It shows direct interaction between miRNA-221-3p and ABHD4 mRNA.

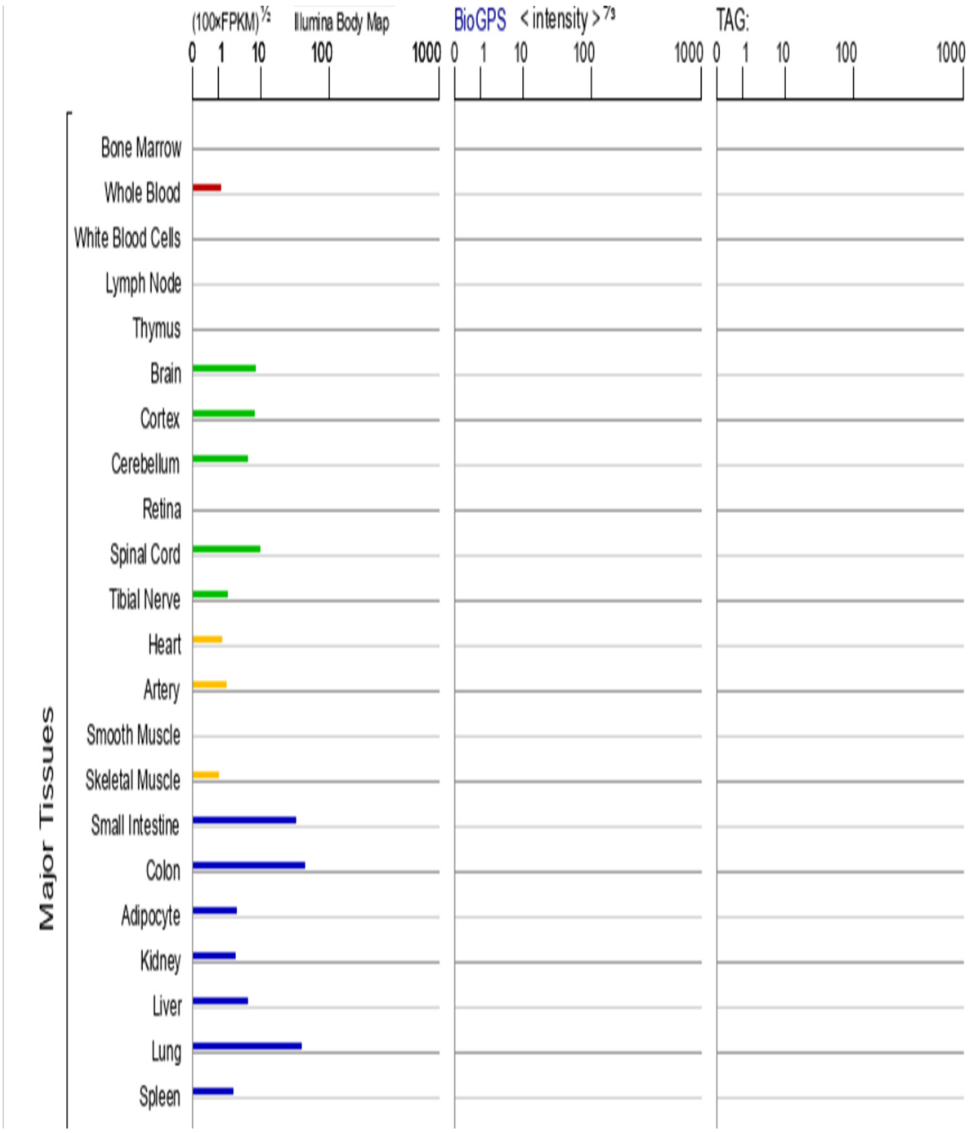

Figure S12. Gene expression of FENDRR lncRNA in heart that was retrieved from GeneCards®: The Human Gene Database. Available at (https://www.genecards.org/cgi-bin/carddisp.pl?gene=FENDRR&keywords=FENDRR,lncRNA, accessed on 1 June 2021) It shows the basal expression of FENDRR lncRNA in normal heart. X-axis represents level of RNA expression while Y-axis represents the tissue in which the gene is expressed.

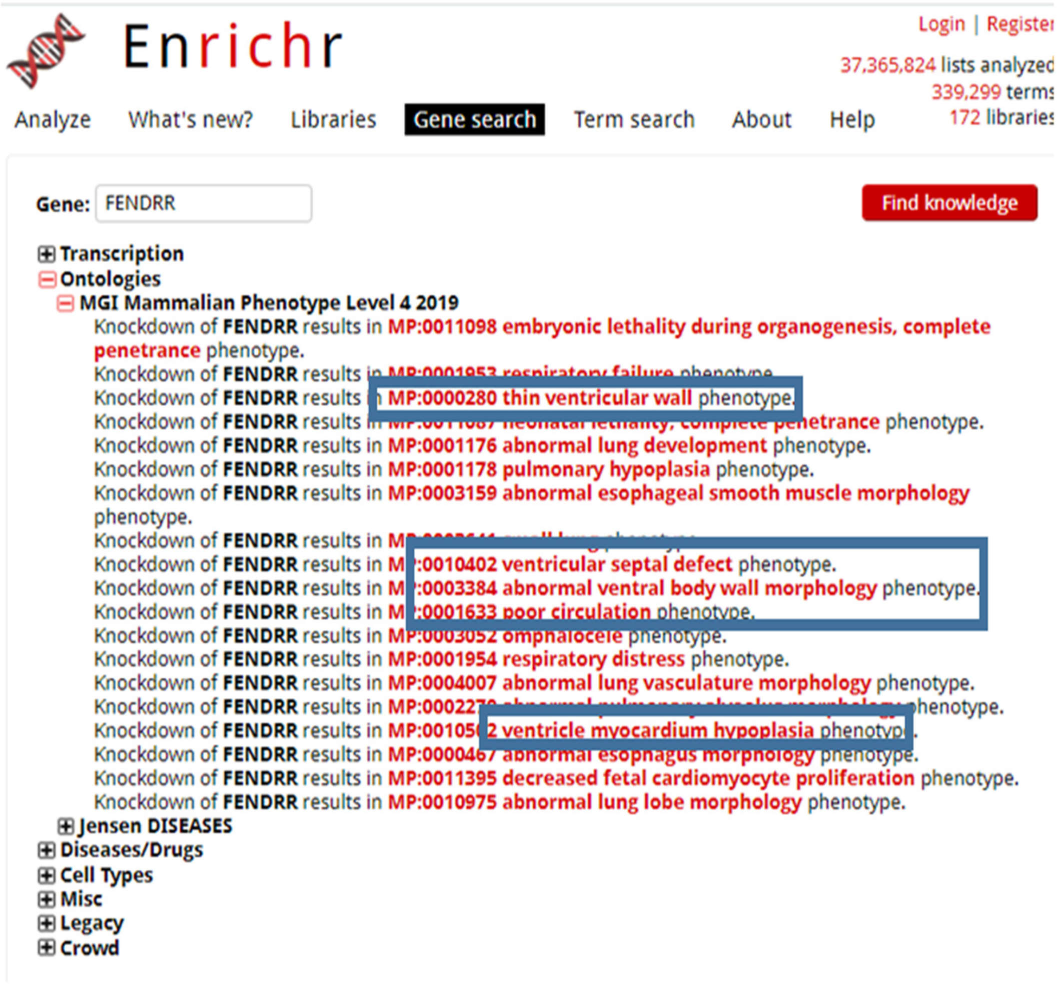

Figure S13. Gene expression of FENDRR lncRNA in heart that was retrieved from Enricher tool database. Available at https://maayanlab.cloud/Enrichr/ (accessed on 1 June 2021). It shows the FENDRR lncRNA relation to different cardiac disorders.

lncipedia.org/db/search?search_id=fendrr

FENDRR:15	FENDRR	chr16:86487716-86490879	-	2825
FENDRR:16	FENDRR	chr16:86487706-86490879	-	2835
FENDRR:17	FENDRR	chr16:86474539-86477993	-	2138
FENDRR:18	FENDRR	chr16:86490183-86508886	-	563
FENDRR:19	FENDRR	chr16:86490268-86508886	-	969
FENDRR:2	FENDRR	chr16:86490268-86508877	-	960
FENDRR:20	FENDRR	chr16:86474529-86508886	-	3121
FENDRR:21	FENDRR	chr16:86474623-86508886	-	3211
FENDRR:22	FENDRR	chr16:86474623-86508886	-	3606
FENDRR:23	FENDRR	chr16:86478011-86508886	-	731
FENDRR:24	FENDRR	chr16:86487737-86508886	-	2630
FENDRR:25	FENDRR	chr16:86473638-86498552	-	4093
FENDRR:26	FENDRR	chr16:86474623-86498552	-	3164

Activate Windows

Figure S14. FENDRR lncRNA detailed isoform that was retrieved from LNCipedia database, (accessed on 1 June 2021). It presents the details of FENDRR lncRNA isoforms.

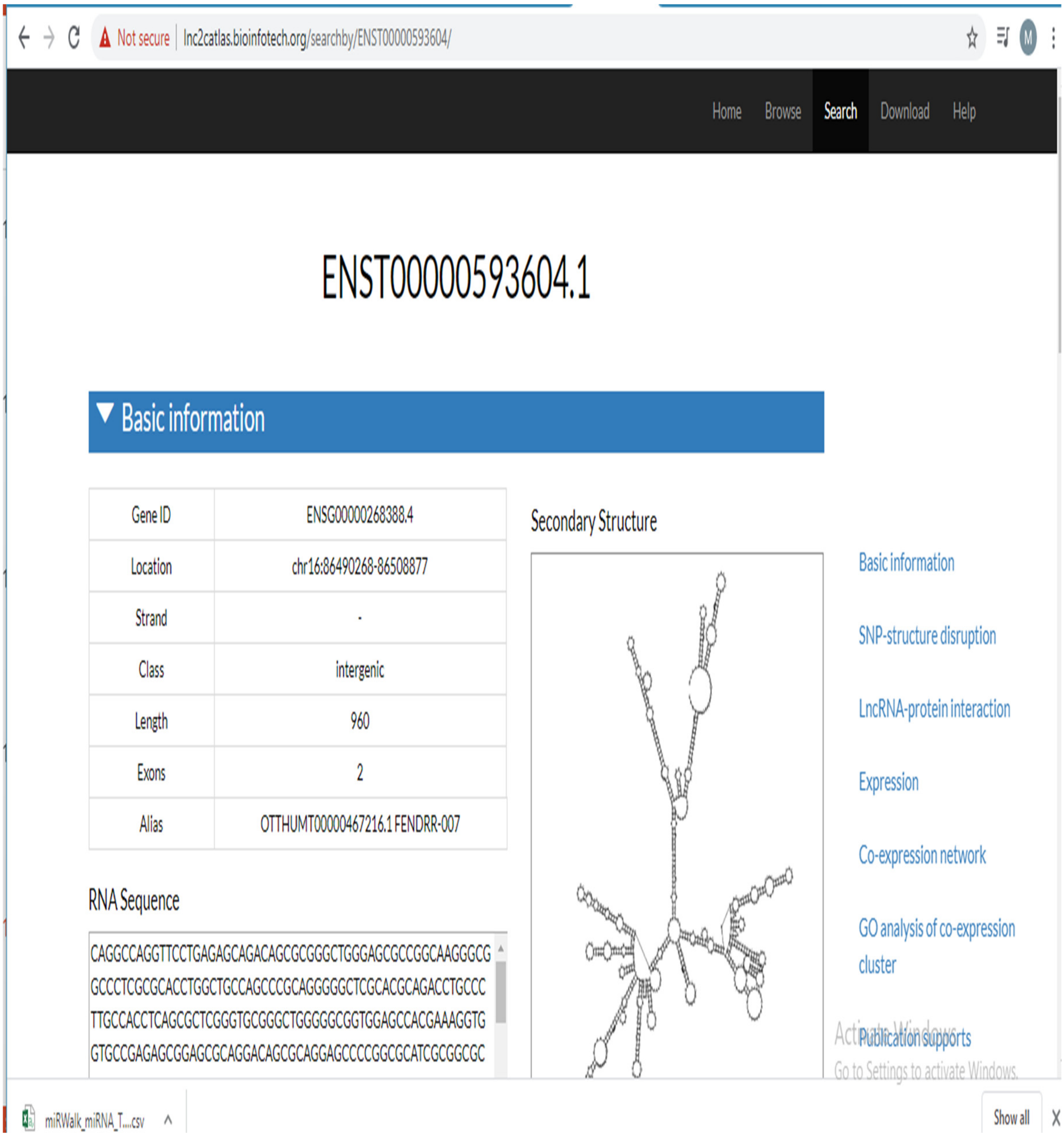

Figure S15. FENDRR lncRNA structure that was retrieved from Lnc2atlas database (accessed on 1 June 2021). It present the detailed structure of FENDRR lncRNA.

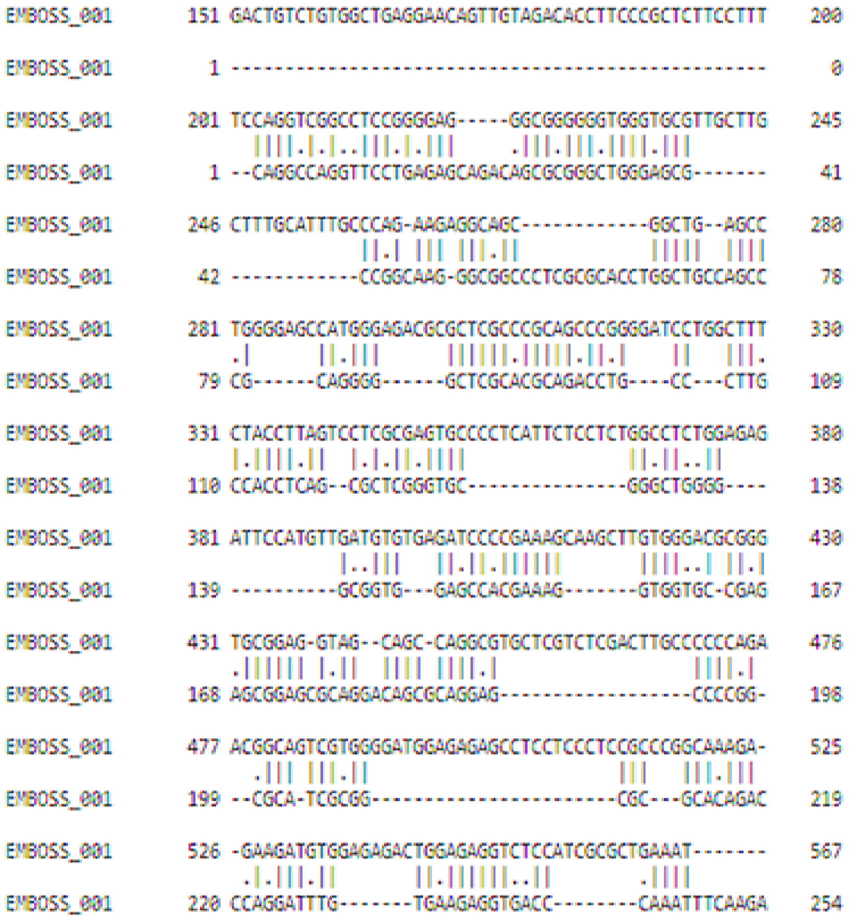

Figure S16. Alignment between ABHD4 mRNA and FENDRR lncRNA that was performed using European bioinformatics institute database (accessed on 1 June 2021). It shows putative interaction between ABHD4 mRNA and FENDRR.

```
EMBOSS_001         1 --------------------------------------------------      0

EMBOSS_001       451 GGCAGATAGGGGTTGGTTATCCTATTGTCTTTACTTTCATATATTTTATT    500

EMBOSS_001         1 --------------------------------------------------      0

EMBOSS_001       501 TTTTCTGTAATAAAACAAATTAAAATCACCTGTCGTGACCTCACAGGGGC    550

EMBOSS_001         1 --------------------------------------------------      0

EMBOSS_001       551 CAGTGTGGGTTGTGGCGTGAGGCAGGAAGCTCTGCAGAGTTCCTTGGTCA    600

EMBOSS_001         1 --------------------------------------------------      0

EMBOSS_001       601 AAAGGATTAATTACAAAAGAACTCCATGAACACCGAGAAGAAGAGAAGGG    650

EMBOSS_001         1 --------------------------------------------------      0

EMBOSS_001       651 AATCCTGGCATTTAAAGCCACAAAGAAACTTGATATGGTCTTGTCTCCT-    699
                                    |||.|||                  ::|:|:.|:
EMBOSS_001         1 ---------------AGCUACA------------------UUGUCUGCUG     17

EMBOSS_001       700 --TCTCTCAATTGACAGTGAGAAACTGAAGACCAGAGAGGGTGAGTGGTT    747
                       :.:|
EMBOSS_001        18 GGUUUC--------------------------------------------     23

EMBOSS_001       748 TACTCAGTTTGGCACAACTGACCCCAACCTAGCCCTCCATGAGGACTGAG    797

EMBOSS_001        24 --------------------------------------------------     23

EMBOSS_001       798 CGCATGAGAGATCCTGAGCCACAGCCGCCCAGCCCTGCTCCTCTCGAATT    847

EMBOSS_001        24 --------------------------------------------------     23

EMBOSS_001       848 TCTGACCTACAGGAACTGCAAGAAGTAATGAAAGACTGCTGTTTAAAGCC    897
```

Figure S17. Alignment between hsa-miRNA-221-3p and FENDRR lncRNA that was performed using European bioinformatics institute database (accessed on 1 Jaunary 2021). It shows putative interaction between hsa-miRNA-221-3p and FENDRR.

Query	Length_Query	Target	Length_Target	dG	ndG	Start_Position_Query	End_Position_Query	Start_Position_Target	End_Position_Target
FENDRR	969	hsa-miR-221-3p	23	-7.25	-0.4833	234	256	1	23

```
Query    Length_Query    Target    Length_Target    dG    ndG    Start_Position_Query    End_Position_Query
Start_Position_Target    End_Position_Target
FENDRR   969   hsa-miR-221-3p   23   -7.25   -0.4833   234   256   1   23
5'
ACTGCGCGCCAGGCCAGGTTCCTGAGAGCAGACAGCGCGGGCTGGGAGCGCCGGCAAGGGCGGCCCTCGCGCACCTGGCTGCCAGCCCGCAGGGGGCTCGCACGCAGACCTGCCCTTGCCACCTCAGCGCTCGGGTGCGGGCTGGGGGCGGTGGAGCCACGAAAGGTGGTGCCGAGAGCGGAGCGCAG
GACAGCGCAGGAGCCCCGGCGCATCGCGGCGCGCACAGACCCAGGATTTGTGAAGAGGTGACCCAAATTTCAAGACAATGTTGGATGCAAACGGATTTGCCAGCAACTGCATCACTGTCAAAACCAGCTCTGCCCGTGTCTTCCGAAGATACCAAGTGAAATACATGTAGATGGGATTTACATCTGAA
AAACCAAGGCAAGAGGCCATGGGAAGGGACGGAGGTGGAACAGGACTGGACAGAAGCTGATAAATGGTTGTTGCTGAGTACTGGGCAGATAGGGGTTGGTTATCCTATTGTCTTTACTTTCATATATTTTATTTTTTCTGTAATAAAACAAATTAAAATCACCTGTCGTGACCTCACAGGGGCCAGTG
TGGGTTGTGGCGTGAGGCAGGAAGCTCTGCAGAGTTCCTTGGTCAAAAGGATTAATTACAAAAGAACTCCATGAACACCGAGAAGAAGAGAAGGGAATCCTGGCATTTAAAGCCACAAAGAAACTTGATATGGTCTTGTCTCCTTCTCTCAATTGACAGTGAGAAACTGAAGACCAGAGAGGGTGAGT
GGTTTACTCAGTTTGGCACAACTGACCCCAACCTAGCCCTCCATGAGGACTGAGCGCATGAGAGATCCTGAGCCACAGCCGCCCAGCCCTGCTCCTCTCGAATTTCTGACCTACAGGAACTGCAAGAAGTAATGAAAGACTGCTGTTTAAAGCCACTGCATTTTGGCATGATTTGTTATGCAGTCGTA
GATAACCAGAAAACATCGGATTTACATTT 3'

.......||.||..||||||||.

3' AGCTACATTGTCTGCTGGGTTTC 5'
```

Figure S18. Alignment between hsa-miRNA-221-3p and FENDRR lncRNA that was performed using LncTar: an efficient tool for predicting RNA targets of lncRNAs (http://www.cuilab.cn/lnctar, accessed on 1 Jaunary 2021). It shows putative interaction between hsa-miRNA-221-3p and FENDRR.

```
EMBOSS_001       1 --CAG-GCCAGGTTCCTGAGAGCAGACAGCGCGGGCTGGGAGCGCCGGCA     47
                      |.| |||.||:    ||||..|.|||       :||||     ||.|
EMBOSS_001       1 GGCUGUGCCGGGU-----AGAGAGGGCAG-------UGGGA-----GGUA     33

EMBOSS_001      48 AGGG-----CGGCCCTCGCGCACCTGGCTGCCAGCCCGCAGGGGGCTCGC     92
                   ||.|     |..||.:|.| ||||: .|: |||.||.|||.||
EMBOSS_001      34 AGAGCUCUUCACCCUUCAC-CACCU-UCU-CCACCCAGCAUGG-------     73

EMBOSS_001      93 ACGCAGACCTGCCCTTGCCACCTCAGCGCTCGGGTGCGGGCTGGGGGCGG    142
                          ||
EMBOSS_001      74 -------CC-----------------------------------------     75

EMBOSS_001     143 TGGAGCCACGAAAGGTGGTGCCGAGAGCGGAGCGCAGGACAGCGCAGGAG    192
```

Figure S19. Alignment between hsa-miRNA-197-5p and FENDRR lncRNA that was performed using European bioinformatics institute database (accessed on 1 Jaunary 2021). It shows putative interaction between hsa-miRNA-197-5p and FENDRR.

Query	Length_Query	Target	Length_Target	dG	ndG	Start_Position_Query	End_Position_Query	Start_Position_Target	End_Position_Target
FENDRR	969	hsa-miR-197-5p	23	-8.44	-1.4067	311	333	1	23

```
Query          Length_Query          Target          Length_Target          dG          ndG          Start_Position_Query          End_Position_Query
Start_Position_Target          End_Position_Target
FENDRR   969   hsa-miR-197-5p   23   -8.44   -1.4067   311   333   1   23
5'
ACTGCGCGCCAGGCCAGGTTCCTGAGAGCAGACAGCGCGGGCTGGGAGCGCCGGCAAGGGCGGCCCTCGCGCACCTGGCTGCCAGCCCGCAGGGGGCTCGCACGCAGACCTGCCCTTGCCACCTCAGCGCTCGGGTGCGGGCTGGGGGCGGTGGAGCCACGAAAGGTGGTGCCGAGAGCGGAGCGCAG
GACAGCGCAGGAGCCCCGGCGCATCGCGGCGCGCACAGACCCAGGATTTGTGAAGAGGTGACCCAAATTTCAAGACAATGTTGGATGCAAACGGATTTGCCAGCAACTGCATCACTGTCAAAACCAGCTCTGCCCGTGTCTTCCGAAGATACCAAGTGAAATACATGTAGATGGGATTTACATCTGAA
AAACCAAGGCAAGAGGCCATGGGAAGGGACGGAGGTGGAACAGGACTGGACAGAAGCTGATAAATGGTTGTTGCTGAGTACTGGGCAGATAGGGGTTGGTTATCCTATTGTCTTTACTTTCATATATTTTATTTTTTCTGTAATAAAACAAATTAAAATCACCTGTCGTGACCTCACAGGGGCCAGTG
TGGGTTGTGGCGTGAGGCAGGAAGCTCTGCAGAGTTCCTTGGTCAAAAGGATTAATTACAAAAGAACTCCATGAACACCGAGAAGAAGAGAAGGGAATCCTGGCATTTAAAGCCACAAAGAAACTTGATATGGTCTTGTCTCCTTCTCTCAATTGACAGTGAGAAACTGAAGACCAGAGAGGGTGAGT
GGTTTACTCAGTTTGGCACAACTGACCCCAACCTAGCCCTCCATGAGGACTGAGCGCATGAGAGATCCTGAGCCACAGCCGCCCAGCCCTGCTCCTCTCGAATTTCTGACCTACAGGAACTGCAAGAAGTAATGAAAGACTGCTGTTTAAAGCCACTGCATTTTGGCATGATTTGTTATGCAGTCGTA
GATAACCAGAAAACATCGGATTTACATTT 3'

.......||||||.......|||

3' GGAGGGTGACGGGAGAGATGGGC 5'
```

Figure S20 Alignment between hsa-miRNA-197-5p and FENDRR lncRNA that was performed using LncTar: an efficient tool for predicting RNA targets of lncRNAs (http://www.cuilab.cn/lnctar, accessed on 1 Jaunary 2021). It shows putative interaction between hsa-miRNA-197-5p and FENDRR.
